# Eight Element Side Edged Framed MIMO Antenna Array for Future 5G Smart Phones

**DOI:** 10.3390/mi11110956

**Published:** 2020-10-24

**Authors:** Saad Hassan Kiani, Ahsan Altaf, Mujeeb Abdullah, Fazal Muhammad, Nosherwan Shoaib, Muhammad Rizwan Anjum, Robertas Damaševičius, Tomas Blažauskas

**Affiliations:** 1 Electrical Engineering Department, City University of Science and Information Technology, Peshawar 25000, Pakistan; saad.kiani@cusit.edu.pk; 2 Electrical Engineering Department, Istanbul Medipol University, Istanbul 34083, Turkey; aaltaf@st.medipol.edu.tr; 3 Department of Computer Science, Bacha Khan University, Charsadda 24420, Pakistan; mujeeb.abdullah@gmail.com; 4 Electrical Engineering Department, University of Engineering and Technology, Mardan 23200, Pakistan; fazal.muhammad@uetmardan.edu.pk; 5 Research Institute for Microwave and Millimeter-Wave Studies, National University of Sciences and Technology, Islamabad 44000, Pakistan; nosherwan.shoaib@seecs.edu.pk; 6 Department of Electronic Engineering, The Islamia University of Bahawalpur, Bahawalpur 63100, Pakistan; engr.rizwan@iub.edu.pk; 7 Department of Software Engineering, Kaunas University of Technology, LT-51423 Kaunas, Lithuania; tomas.blazauskas@ktu.lt

**Keywords:** MIMO antenna, 5G, mobile terminals, Liquid Crystal Display (LCD), smart phone, smart city

## Abstract

This paper presents a novel design of a Multiple Input Multiple Output (MIMO) antenna system for next generation sub 6 GHz 5G and beyond mobile terminals. The proposed system is composed of a main board and two side boards. To make the design cost-effective, FR4 is used as a substrate. The design is based on a unit monopole antenna etched at the side substrate. The single element is resonating at 3.5 GHz attaining a 10 dB bandwidth of 200 MHz and a 6 dB bandwidth of 400 MHz. The single element is then transformed into an MIMO array of 8-elements with an overall dimension of 150 mm × 75 mm × 7 mm, providing pattern diversity characteristics and isolation better than −12 dB for any two radiating elements. A number of studies such as effects of human hand on the system that includes single hand mode and dual mode scenarios and the effects of Liquid Crystal Display (LCD) over the principal performance parameters of the system are presented. The envelop correlation coefficient (ECC) is computed for all the scenarios and it is found that ECC is less than 0.1 for any case and maximum channel capacity is 38.5 bps/Hz within the band of interest. The main advantage of the proposed design over available designs in the literature is that almost all of the main substrate is empty providing wide space for different sensors, systems, and mobile technology components. A brief literature comparison of the proposed system is also presented. To validate the proposed model, a prototype is fabricated and results are presented. This design can be applied on higher frequencies to future micromachines for on chip communications using same theocratical approach as the space for higher frequencies in mmwave spectrum has been reserved. The simulated results are in an excellent agreement with the measured results. All the main performance parameters of the design are calculated and compared with the measured results wherever possible.

## 1. Introduction

Currently, researchers and developers face rising challenges in creating the Internet-of-Things (IoT)-based systems that can be smoothly integrated with the 5th generation (5G) communications [1]. Multiple Input Multiple Output (MIMO) antenna systems for 5G wireless communication are considered to be one of the key technologies for IoT and smart city infrastructures [2]. They are widely researched and focused in order to be deployed on large scale on the latent half of 2020. MIMO technology enables higher data rate with lower latency and channel capacity as compared to its predecessor 4G/4G Long-Term Evolution (LTE) communication standards. Multiple antenna element (AEs) system assures all the elements work simultaneously providing maximum gain, low envelope correlation coefficient (ECC), and pattern diversity [3]. Current MIMO systems support the number of AEs up to four in a single package [4,5,6]. In 5G MIMO systems, more than or a minimum of six AEs are desired to be installed in order to meet the requirements of transmission rate in order to satisfy the fixed communication bandwidth and higher interference resistance conditions. Although, a large number of studies have shown that usually eight element arrays are frequently proposed for 5G MIMO systems. The number of AEs up to ten [7,8], twelve [9] even up to 18 elements [10], have also been published for enhanced channel capacity although such rise in AEs introduces system complexity and stability factors. Designing an MIMO system for 5G mobile terminals is a challenging task since the arrangement of AEs in a small space can give rise to poor isolation which could alter system performance characteristics on the chassis [11]. The efficient techniques reported for isolation enhancement are meander line resonators, defected grounds, and electromagnetic bandgap structures and also through parametric spacing among radiating elements. Reportedly, several studies and MIMO AEs have been developed in [12,13,14,15,16,17] consisting of slots antennas, planner inverted-F antennas, slotted monopoles, and loop elements.

While using eight elements, the maximum channel capacity increases up to 36 bps/Hz roughly eight times the standard single element capacity which, with a 200 MHz bandwidth, can result in a data rate much more than 1 Gigabyte/sec as compared to its predecessor with an ideal range of 18 bps/Hz over 20 dB SNR levels. In [18], an eight-element diamond ring dual polarized slot antenna has been proposed with maximum gain up to 3 dB and ECC less than 0.01 for any two AEs. In [19], an eight-port antenna has been reported with four AEs edged near the end of chassis and four vertically mounted in the middle side part offering high bandwidth with channel capacity (CC) of 34.25 bps/Hz and ECC less than 0.16. With high large ground clearance, an isolation of 10 dB is reported using orthogonal mode pair in [20]. A monopole self-isolated U shape antenna fed by a meandered line is presented in [21]. It is based on curved side frames covering the N77 band based on −6 dB criteria with ECC less than 0.08 and channel capacity of 35 bps/Hz. However, curved frames increase complexity with higher stability issues. In [22], the multiple antenna elements array up to sixteen AEs is obtained by accommodating decoupled printed open-end slot antennas positioned along two antipodal side edges of the chassis. Placing AEs on the corner of the circuit board in a pair of duos perpendicular to each other [23] exhibited dual polarized response due to their orthogonal placement with higher channel capacity and low ECC; but, such arrangement on planner surface alongside the length of chassis may worsen the signal integrity due to closeness with other electronic components mounted in the middle and bottom side of the ground plane.

In this paper, an eight element MIMO array structure is presented. The proposed system covers the frequency range between 3.4 GHz to 3.6 GHz with less intricacy. It is helpful to use intra-band contiguous carrier aggregation (IBCCA) to surge the data throughput. Such a solution can be applied to a future 5G smart phone. The proposed antenna system is designed in such a manner that the insulation between the radiating elements are below −12 dB without using any decoupling structure. In addition, the elements are etched on the sides to provide space for other electronic components. The MIMO antenna array performance parameters are also evaluated with user hand scenarios and liquid crystal display (LCD).

## 2. Antenna Design

**Figure 1:**
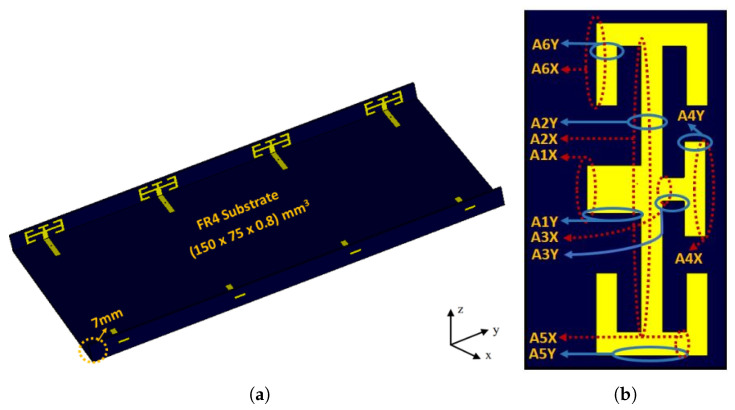
(**a**): Perspective view of the proposed Multiple Input Multiple Output (MIMO) system, (**b**): single monopole antenna element.

The perspective view of the proposed MIMO array design is shown in Figure 1a. It is composed of eight elements, printed on a commercially available FR4 substrate with a relative permittivity of 4.4, and substrate thickness of 0.8 mm.

The proposed AEs consists of one main substrate and two side substrates. The overall dimension of the main substrate is 150 mm × 75 mm × 0.8 mm, whereas for side substrates the height of the substrate is chosen such that it can be used in the modern mobile phones slim structures. The height of the side substrates is 7 mm. The ground plane from both upper and lower edges have been reduced to 3 mm for ground clearance. The radiating elements are printed on side substrates while the feeds are printed on the main substrate. Table 1 shows the detail dimensions of unit antenna.

### Design Evolution

**Figure 2:**
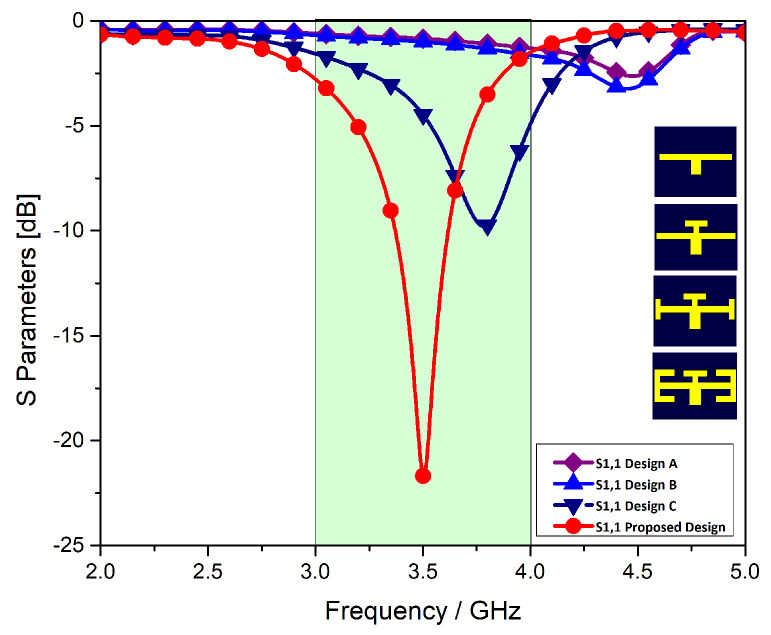
Return loss of different antenna designs.

In this paper, we describe the design of an MIMO antenna system that resonates around 3.5 GHz. To achieve the desired response, the proposed single monopole antenna element is a result of modification of series of structures composed of different stubs. The geometric parameters of the stubs are optimized. The response of each stage and/or design within the band of interest is shown in Figure 2.

**Figure 3:**
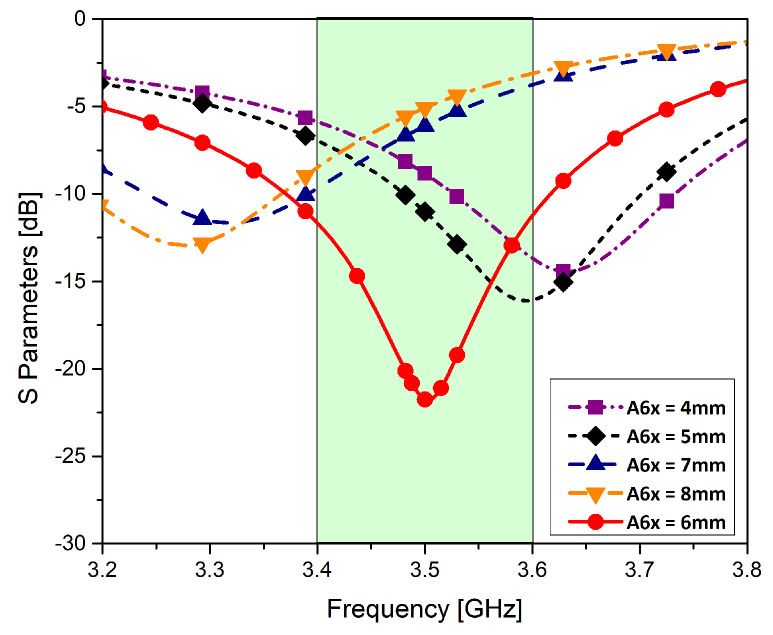
Effects of A6x on the reflection coefficient of a single element.

In the first stage, a T-shape was etched which produced a spike response at 4.5 GHz. In stage-2, two horizontal stubs are introduced to the previous T-shaped design. A slight shift in the resonant frequency is obtained in stage-2. In the next stage, two I-shaped strips were added to the T-shaped structure to shift the resonant frequency towards the desired frequency band. This results in the shift of resonant frequency from 4.5 GHz to 3.9 GHz. The proposed element is designed in the final stage by introducing two rotated T-shapes at the either side of the previous design. The dimensions of the final design are detailed in Table 1. The reflection coefficient of the proposed single monopole antenna element is shown in red color in the Figure 3. It is clear from the figure that the structure is resonating at 3.5 GHz with a 10 dB bandwidth of 200 MHz and 6 dB bandwidth of 500 MHz. The unit monopole is tuned by parameter A6X in order to achieve the central frequency of 3.5 GHz as shown in Figure 3.

## 3. Results and Discussion

**Figure 4:**
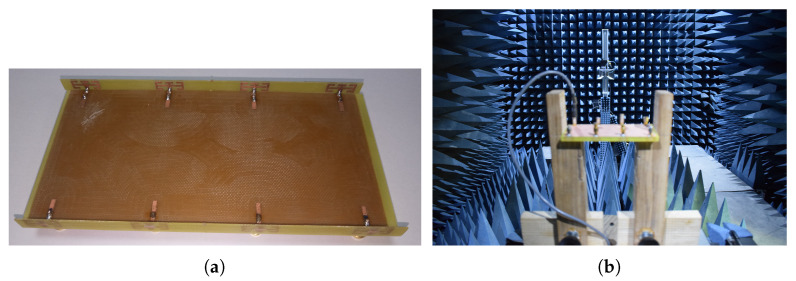
(**a**) Fabricated prototype front view, (**b**) far-field measurement set up.

The proposed 8-element MIMO antenna system is designed and simulated in CST 2020. The model is fabricated using the LPKF machine.The fabricated model is shown in the Figure 4a and the farfield measurement set up is shown in Figure 4b. The measurements were done by an Agilent (Keysight Technologies, Santa Rosa, CA, USA) vector network analyzer.

### 3.1. S Parameter Analysis

**Figure 5:**
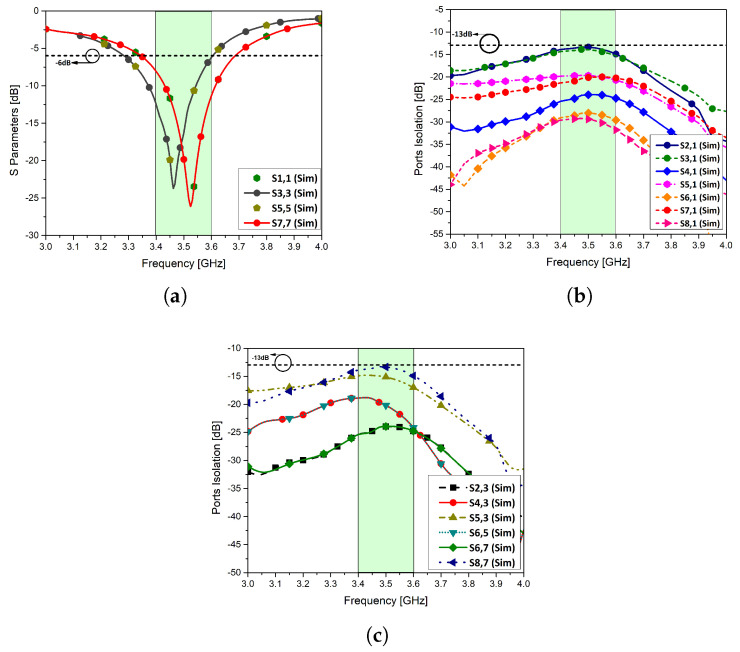

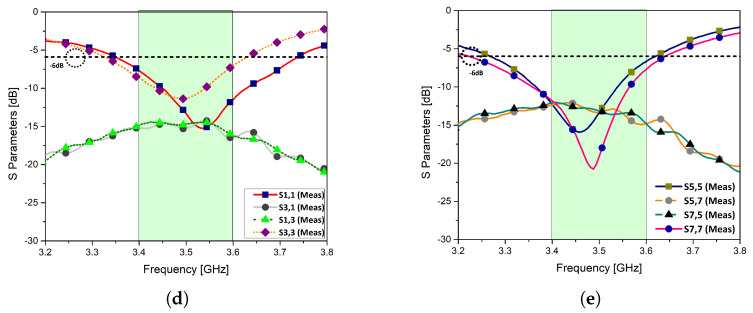
(**a**): Reflection coefficient of proposed MIMO configuration, (**b**): port isolation of antenna element 1, (**c**): port isolation of selected antenna elements, (**d**): measured S-parameters of antenna elements 1 and 3, (**e**): measured S-parameters of antenna elements 5 and 7.

The proposed design is assembled in an MIMO configuration of eight elements with four elements on either sides with edge to edge distance of 28 mm, nearly half wavelength apart. Due to symmetry of the structure, a side of the proposed model is discussed. The computed reflection coefficient of antenna elements 1, 3, 5, and 7 is shown in Figure 5a.

The port isolation between antenna element 1 and the others is given in Figure 5b. The isolation of antenna element 1 with the radiating elements in a close proximity is below −13 dB for the entire band of interest and for the remaining antenna elements, it is lower than −20 dB. The isolation characteristics for antenna elements 3, 5, and 7 are plotted in Figure 5c. The studies show that all radiating elements shows a good isolation of at least −13 dB. For antenna elements that are far from each other, the isolation is less than −25 dB. The measured results for antenna elements 1, 3, 5, and 7 are shown Figure 5d,e. The measured results are in well agreement with the simulated results. For isolation characteristics of the system, antennas in the close proximity are chosen to validate the simulated results. It can be seen from the results that isolation between the antennas is below −13 dB.

### 3.2. Efficiencies

**Figure 6:**
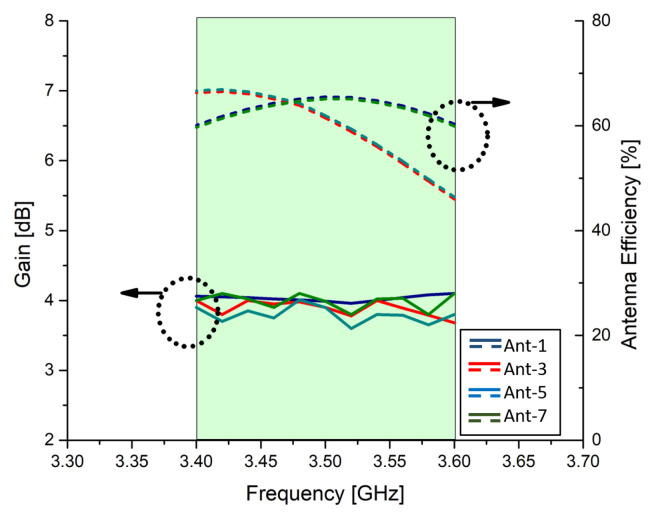
Total efficiency and maximum gain over frequency of MIMO antenna system.

The total efficiency of the proposed MIMO antenna and gain over the band of interest is given in Figure 6. The total efficiency of the antenna elements 3 and 5 ranges from 45% to 70% and 58% to 62% for antenna elements 1 and 7, respectively. The lower efficiencies of the middle AEs can be attributed to the propagation area since the radiating elements on the end of the chassis have free propagating path while the AEs on the middle have propagating elements on both sides. The gain of the proposed antenna varies from 3.5 dB to 3.9 dB, hence providing nearly uniform gain in the desired band.

### 3.3. Radiation Pattern

**Figure 7:**
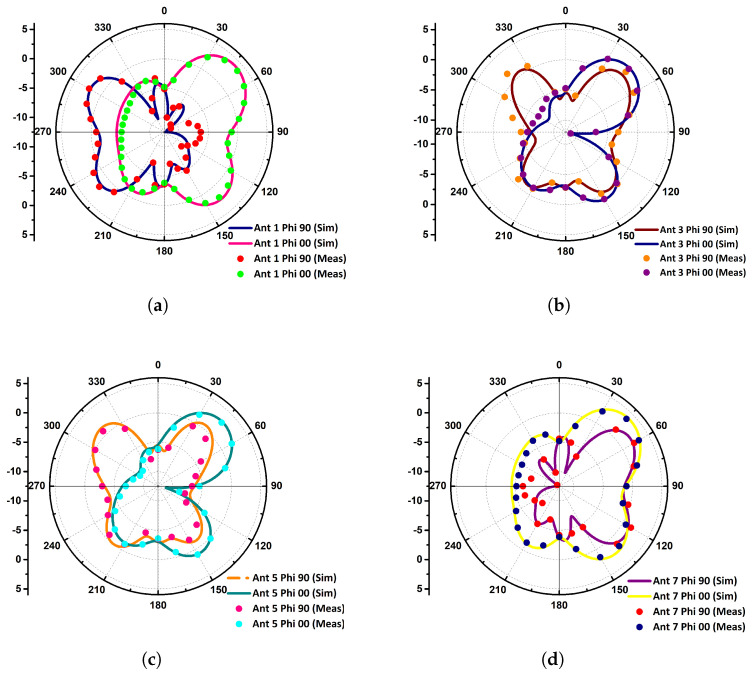
Simulated and measured radiation patterns for (**a**) antenna element (AE)-1, (**b**) AE-3, (**c**) AE-5, and (**d**) AE-7.

The radiation patterns for xz- and yz-plane are shown in Figure 7. The two 1×4 arrays at the either sides have similar radiation characteristics, therefore the radiation performance of a single side is presented. In the proposed antenna system, Ant-1 and Ant-7 are at the corners exhibiting similar far-field patterns. Similarly, Ant-3 and Ant-5 are surrounded by Ant-1 and Ant-7, exhibiting the same radiation characteristics. Ant-1 and Ant-7 have directive radiation patterns for the xz-plane with a maximum at $\phi =0^{\circ }$, $\theta =45^{\circ }$, while the yz-plane patterns are conical, radiating at the either side of the plane. On the other hand, the radiation characteristics of the Ant-3 and Ant-5 are directive for both xz- and yz-planes with a null in the broad side $\phi =0^{\circ }$, $\theta =90^{\circ }$. The radiation patterns as shown in the figure cover complementary space regions; hence, providing pattern diversity characteristics.

### 3.4. MIMO Parameters

**Figure 8:**
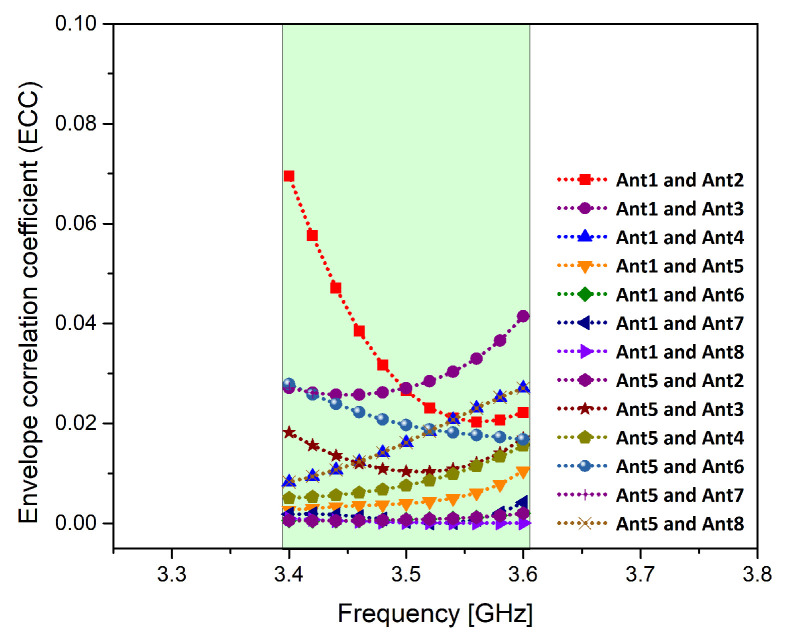
Envelope correlation coefficient (ECC) of the proposed MIMO antenna system.

Envelop correlation coefficient (ECC) is an important MIMO parameter. ECC is a measure of the effect of a radiating element on the performance of the other radiating elements. Low ECC among MIMO elements is necessary to commend the sturdiness of the MIMO system. The ECC of the proposed MIMO antenna using (1) mentioned in [24,25] among any two radiating elements based on far field results is less than 0.1 within the band of interest as shown in Figure 8.

(1) $$ECC=\frac{|{\;\int \int \;}_{4\pi }\left(\vec{B_{i}}\left(\theta ,\phi \right)\right)\times \left(\vec{B_{j}}\left(\theta ,\phi \right)\right){\;d\Omega |}^{2}}{{\;\int \int \;}_{4\pi }|\left(\vec{B_{i}}\left(\theta ,\phi \right)\right){|}^{2}\;d\Omega {\;\int \int \;}_{4\pi }{\left|\left(\vec{B_{j}}\left(\theta ,\phi \right)\right)\right|}^{2}\;d\Omega }$$

where $\vec{B_{i}}\left(\theta ,\phi \right)$ denotes the 3D radiation pattern upon excitation of the *i*th antenna and $\vec{B_{j}}\left(\theta ,\phi \right)$ denotes the 3D radiation pattern upon excitation of the *j*th antenna. Ω is the solid angle.

The Mean Effective Gain (MEG) of the proposed antenna system in order to satisfy the MIMO performance with good channel characteristics is mentioned in Table 2. The MEGs are calculated using (2) mentioned in [4] based on the measured results of 2-D far field and meeting the requirement of $MEG_{i}$≈$MEG_{j}$.

(2) $$MEG=\int_{-\pi }^{\pi }\int_{0}^{\pi }\left[\frac{r}{r+1}G_{\theta }\left(\theta ,\phi \right)P_{\theta }\left(\theta ,\phi \right)+\frac{1}{1+r}G_{\phi }\left(\theta ,\phi \right)P_{\phi }\left(\theta ,\phi \right)\right]sin\theta d\theta d\phi$$

where $G_{\phi }\left(\theta ,\phi \right)$ and $P_{\theta }\left(\theta ,\phi \right)$ are angle of arrival and *r* is the cross polar ratio which can be expressed as Equation (3).

(3) $$r=10log_{10}\left(\frac{P_{vpa}}{P_{hpa}}\right)$$

where the power received by vertically polarized antenna and horizontally polarized antenna are represented as $P_{vpa}$ and $P_{hpa}$, respectively.

**Figure 9:**
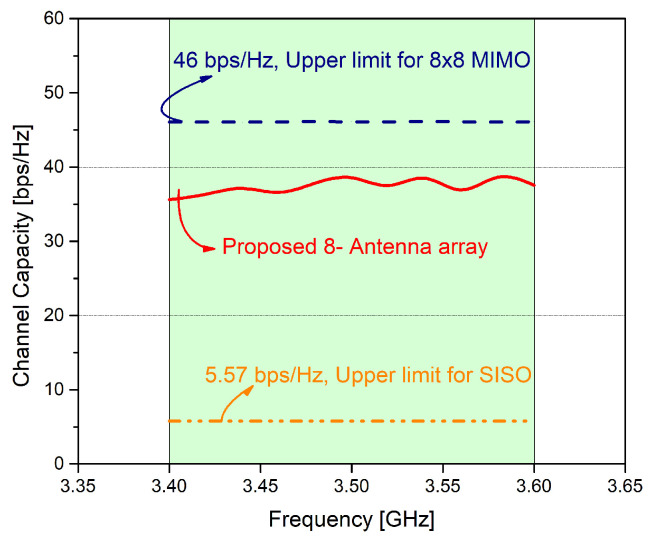
Channel capacity of the proposed MIMO antenna system.

The channel capacity of the MIMO antenna is calculated using equations given in [26]. The derived channel capacity of the eight port proposed MIMO antenna is 38.5 bps/Hz as shown in Figure 9. From the figure, we can see that the calculated channel capacity is near to the ideal case of the 8 element MIMO system, i.e., 46 bps/Hz, and is sufficient enough to deliver optimum performance in MIMO systems.

### 3.5. User Hand Effects

**Figure 10:**
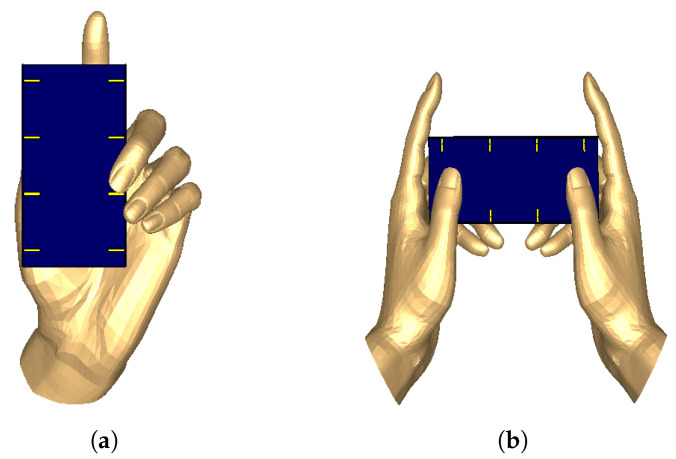
User hand influence of (**a**): single hand mode (SHM) and (**b**): two hand mode (THM).

With the advancement of technology, personal computers are replaced by the smart phones. The smart phones are used for many purposes such as gaming, chatting, and many more. In this study, we analyze single hand mode (chatting) and two hand mode (gaming) scenarios. This additional step is carried out to study the influence of human body interaction on the performance of a mobile terminal antennas. In the simulation model, a human hand phantom model is used. Typically, single hand mode (SHM) and two hand mode (THM) are shown in Figure 10. As 5G mobile terminals are mainly concerned for data transmission, it is necessary to study the effect of user hand.

**Figure 11:**
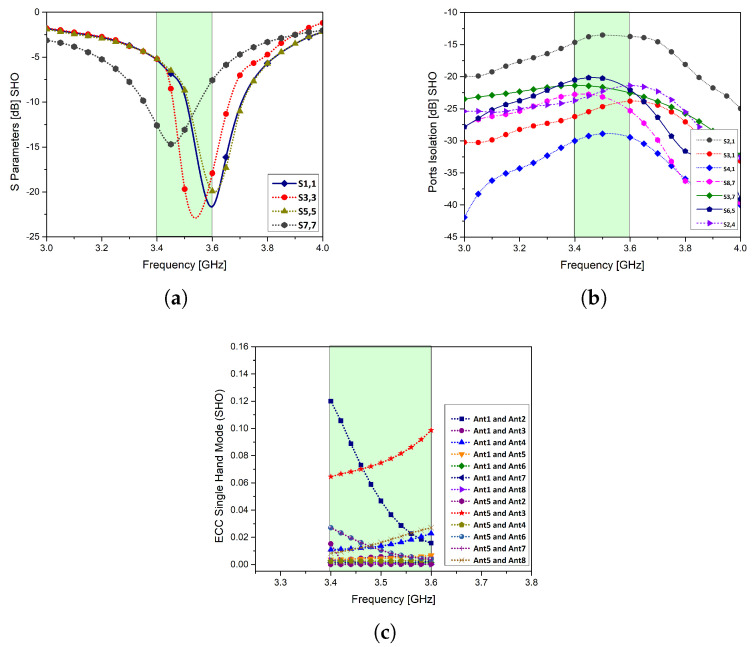
Performance parameters in single hand mode (SHM): (**a**): S-parameters, (**b**): port isolation, (**c**): ECC.

The return loss of different antenna elements is shown in Figure 11a,b. In SHM, antenna elements 3 and 5 are in a direct contact with the phantom hand; therefore, the reflection coefficient has shifted slightly which can be attributed to the absorption losses, causing slight frequency detuning. However, the port isolation is still better than −14 dB ensuring smooth operation. The ECC of MIMO antenna elements for the SHM analysis is less than 0.14 within the band of interest as shown in Figure 11c.

**Figure 12:**
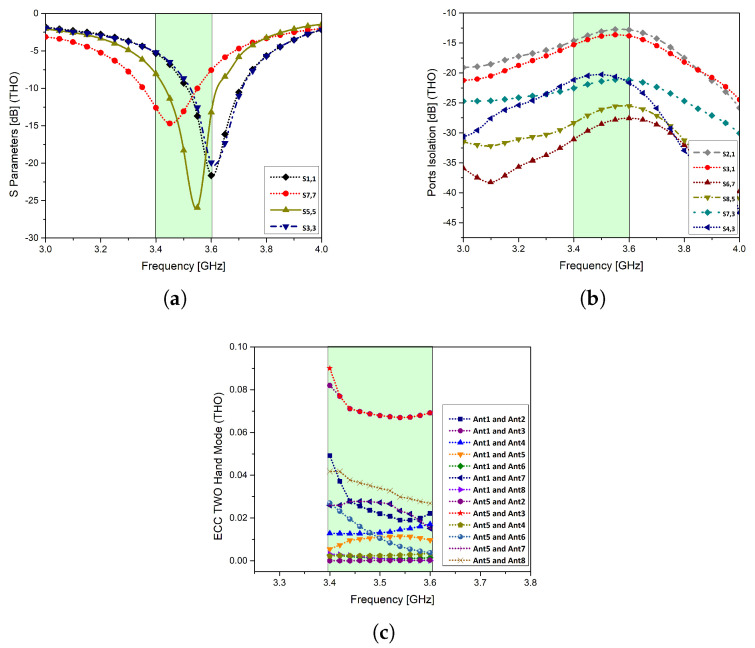
Performance parameters in two hand mode (THM): (**a**): S-parameters, (**b**): port isolation, (**c**): ECC.

In THM, the thumbs are on the main substrate, while the side substrates are grasp by fingers covering antenna elements 1 and 7 completely. Since the material that defines the hand is assumed to be lossy, the return loss response of antenna elements 1 and 7 are shifted by 1 GHz. However, the isolation between the radiating elements is lower than −12 dB and ECC is less than 0.10 throughout the band of interest (Figure 12).

### 3.6. Liquid Crystal Display (LCD)

**Figure 13:**
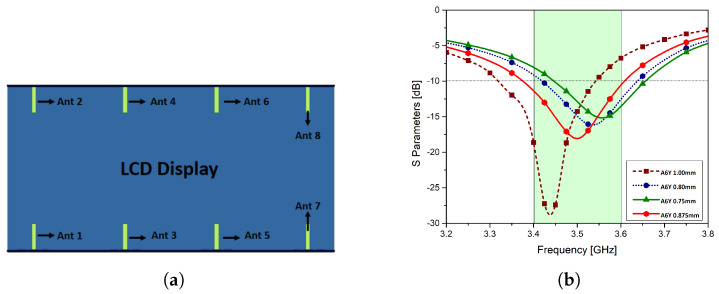
(**a**): Liquid crystal display (LCD) display module with the proposed MIMO antenna system, (**b**): parametric tuning of parameter A6x.

The effect of liquid crystal display (LCD) on the key performance parameters of the MIMO array system is discussed in this section. The module is assumed to be of 1 mm in thickness with a dielectric constant of 7 and loss tangent of 0.02. The dimension of the LCD is 150 mm × 75 mm as shown in the Figure 13a. The LCD display is lying on the two side substrates. The main substrate is under the display and the side substrates are at the edges of the LCD. This scenario is used in the study by the Cellular Telecommunication Industry Association (CTIA) standards version 3.4 for 5G mobile terminals. Due to dielectric loading, the return loss has been shifted slightly, however, still fulfilling the voltage standing wave ratio (VSWR) 2:1 criteria. The return loss response of the proposed MIMO system can be parametrically adjusted by slightly varying the monopole parameter A6× as shown in Figure 13b. By tuning the proposed monopole parameter, the central frequency of 3.5 GHz is achieved at the value 0.875 mm.

The proposed MIMO antenna design can be improved by deploying decoupling network between the radiating elements to reduce mutual coupling. The antennas are designed on the sides which make the system slightly complex. To improve the design and make it suitable for future technologies, few studies are under consideration. One possible improvement will be use of decoupling network. Another improvement will be to modify the response to resonate at different frequencies to cover wide range of technologies. This work has many advantages over the studies available in the literature. Therefore, a detailed literature comparison with the proposed work is presented in Table 3. In all these studies, an 8-element system is considered for comparison. It is evident that the proposed work is an improved and enhanced work for future 5G mobile terminals.

## 4. Conclusions

In this paper an eight element MIMO antenna system is proposed for future 5G smart phones and future technologies. Each antenna element operates between 3.4 GHz to 3.6 GHz with a more than −13 dB port isolation and with ECC less than 0.1 among any two radiating elements. The system provides an excellent pattern diversity within the band of interest. The MIMO parameters are computed and presented. The proposed system’s MEGs cover the required standard and channel capacity is found to be at 38.5 bps/Hz, which is seven times larger than ideal Single Input Single Output (SISO)systems capacity of 5.7 bps/Hz. The proposed antenna is designed, fabricated, and measured. The simulated and measured results are in an excellent agreement. To analyze, study, and understand the performance of the proposed system, practical scenarios such as user hand analysis and LCD display is also investigated. The proposed antenna system is simple, efficient, and provide good port isolation, higher efficiencies, and other MIMO parameters without degrading the key performance of the system.The main advantage of the proposed design over available designs is that almost all of the main substrate is empty providing wide space for different sensors, mmwave mobile technology components and future micromachines since using the same methodology the and theoretical approach the design can be implemented for microwavelength systems and components. Thus it can be concluded that it can be used as a potential candidate for future mobile terminals and smart cities.

## Figures and Tables

**Table 1:** Dimensions of the proposed unit monopole element.

Component	Value (mm)	Component	Value (mm)
A1X	2	A1Y	2.5
A2X	1	A2Y	14
A3X	1	A3Y	1.1
A4X	1	A4Y	4
A5X	1	A5Y	6
A6X	6	A6Y	1

**Table 2:** Calculated Mean Effective Gain (MEG) values of the MIMO Antenna.

Frequency	MEG1	MEG2	MEG3	MEG4	MEG5	MEG6	MEG7	MEG8
3.5GHz	−2.96	−2.98	−3.02	−3.13	−3.09	−2.84	−2.73	−2.80
Indoor	−4.98	−5.02	−5.45	−5.89	−6.23	−6.15	−5.43	−6.31

**Table 3:** Comparison of the proposed MIMO antenna design with other designs known from the literature.

Refs	Bandwidth	Elements	Unit Size	Antenna	Channel	ECC
	(GHz)		in mm (LxW)	Efficiency	Capacity	
[18]	3.4–3.6	8	5.25 × 3.9	60–80	NA	0.1
	(−10 dB)		(Planar)			
[23]	3.4–3.6	8	10 × 2.5	40–60	37	0.2
	(−10 dB)		(Planar)			
[22]	3.4–3.6	8	3 × 8	30–52	37	0.3
	(−6 dB)		(Planar)			
[27]	3.4–3.6	8	16 × 5	40–52	35	0.15
	(−6 dB)		(Side-Edged)			
[28]	3.4–3.6	8	12 × 12.5	58–72	15	NA
	(−6 dB)		(Planar)			
[29]	3.3–3.6	8	10 × 4.3	40–60	37	0.1
	(−10 dB)		(Side-Edged)			
[30]	3.4–3.6	8	14.2 × 9.4	30–50	NA	0.2
	(−6 dB)		(Planar)			
[31]	3.4–3.6	8	17.4 × 6	50–60	NA	0.15
	(−10 dB)		(Side-Edged)			
Proposed	3.25–3.65	8	14 × 6	58–72	38.5	0.1
	(−6 dB)		(Side-Edged)			
